# Filamentation in *Candida auris*, an emerging fungal pathogen of humans: passage through the mammalian body induces a heritable phenotypic switch

**DOI:** 10.1038/s41426-018-0187-x

**Published:** 2018-11-28

**Authors:** Huizhen Yue, Jian Bing, Qiushi Zheng, Yulong Zhang, Tianren Hu, Han Du, Hui Wang, Guanghua Huang

**Affiliations:** 1 State Key Laboratory of Mycology, Institute of Microbiology, Chinese Academy of Sciences, Beijing, 100101 China; 2 University of Chinese Academy of Sciences, Beijing, 100049 China; 3 State Key Laboratory of Genetic Engineering, School of Life Sciences, Fudan University, Shanghai, 200438 China; 4 Institutes of Biomedical Sciences, Fudan University, Shanghai, 200032 China; 5 Department of Clinical Laboratory, Peking University People’s Hospital, Beijing, 100044 China

## Abstract

Morphological plasticity has historically been an indicator of increased virulence among fungal pathogens, allowing rapid adaptation to changing environments. *Candida auris* has been identified as an emerging multidrug-resistant human pathogen of global importance. Since the discovery of this species, it has been thought that *C. auris* is incapable of filamentous growth. Here, we report the discovery of filamentation and three distinct cell types in *C. auris*: typical yeast, filamentation-competent (FC) yeast, and filamentous cells. These cell types form a novel phenotypic switching system that contains a heritable (typical yeast-filament) and a nonheritable (FC-filament) switch. Intriguingly, the heritable switch between the typical yeast and the FC/filamentous phenotype is triggered by passage through a mammalian body, whereas the switch between the FC and filamentous phenotype is nonheritable and temperature-dependent. Low temperatures favor the filamentous phenotype, whereas high temperatures promote the FC yeast phenotype. Systemic in vivo and in vitro investigations were used to characterize phenotype-specific variations in global gene expression, secreted aspartyl proteinase (SAP) activity, and changes in virulence, indicating potential for niche-specific adaptations. Taken together, our study not only sheds light on the pathogenesis and biology of *C. auris* but also provides a novel example of morphological and epigenetic switching in fungi.

## Introduction

Phenotypic plasticity is a common strategy used by microbial pathogens to adapt to diverse host environments^1–4^. Cells with a unique phenotype often have advantages in specific ecological niches. Transitions between different phenotypic forms allow cells to rapidly respond to environmental fluctuations. Uropathogenic *Escherichia coli* and *Flectobacillus spp*. are capable of altering their cellular morphologies to a filamentous phenotype in response to environmental stress^3^. *Cryptococcus neoformans* and *Saccharomyces cerevisiae* alternate between single-celled yeast and multicellular hyphal or pseudohyphal forms^5–7^. Among common adaptation switching systems, morphological transitions in pathogenic *Candida spp*. are perhaps the best example. Two typical morphological switching systems, namely, yeast-filament transition and white-opaque switching, have been well investigated in *Candida albicans*, a major human fungal pathogen^4,8,9^.

In *C. albicans*, the switch between the normal yeast state and the filamentous form has been well-documented as a response to environmental changes^9,10^. Several host-related factors, such as physiological temperature (37 °C), neutral pH, elevated CO_2_ levels, and serum presence, act as stressors and promote the development of filaments; low temperatures, acidic pH, and rich nutritional conditions promote stabilization of the normal yeast phenotype^10^. Persistence of the filamentous phenotype is highly dependent on external environmental factors. Filamentous *C. albicans* cells quickly switch back to the normal yeast form when the inducing factors are removed from the culture conditions. In contrast, the white and opaque phenotypes are heritable and epigenetically regulated; however, the switching frequencies are affected by environmental factors^11–14^. Both white and opaque cells can heritably maintain their cellular phenotypes for multiple generations.

These morphological transitions are associated with changes in antifungal resistance, virulence, and sexual reproduction across *Candida* species^4,10,12–14^. For example, filamentous cells are better at invading host tissues and are essential for initiating systemic infections, whereas normal yeast cells are more easily disseminated to different organs through the circulatory system. White cells are regarded as more virulent in systemic infection models, and opaque cells are more effective at colonizing skin or cutaneous tissues^13,14^.

The multidrug-resistant fungus *Candida auris* was first reported in Japan in 2009 and has been identified as a globally emerging pathogen^15–17^. *C. auris* infections have been reported in at least 20 countries across five continents^18,19^. Recently, several large-scale nosocomial outbreaks have been reported^20–22^. As a result, *C. auris* has attracted attention worldwide despite the limited biological knowledge base. Since its original characterization in 2009, it was accepted that *C. auris* could not undergo filamentation^15,19,23^. In this study, a filamentous cell type and a novel form of phenotypic switching system were identified and described in *C. auris*. We further demonstrated that passage through a mammalian host via systemic infection efficiently induces this typical yeast-to-filament transition. Further, *C. auris* filamentous cells switched to a filamentation-competent (FC) yeast form following growth at host physiological temperature or under in vivo conditions; however, the filamentation capacity was maintained in FC yeast cells. Global gene expression and virulence assays further supported the presence of typical yeast and filamentous cell phenotypes as distinct features, improving the overall understanding of species-specific virulence.

## Results

### Passage through a mammalian body triggers a filamentous phenotype

It has long been thought that *C. auris* was unable to form germ tubes or pseudohyphae^19,24^. *C. auris* is a member of the CTG clade, which includes species that translate the CTG codon as serine instead of leucine^25^. Many of these species, including *C. albicans*, *Candida tropicalis*, and *Candida haemulonii*, can produce hyphae or pseudohyphae in response to environmental changes^8,24^. A variety of environmental factors, including presence of serum, N-acetylglucosamine (GlcNAc) exposure, and high CO_2_ levels, are potent inducers of *C. albicans* filamentation^10^. The effects of these inducers on the development of filaments in *C. auris* were tested at both 25 and 37 °C. Under these in vitro culture conditions, no filamentous growth was observed in *C. auris*^26^. Intriguingly, development of a pseudohyphal-like phenotype was observed when *C. auris* cells were treated with 10% NaCl^26^, suggesting that this species has the potential to undergo filamentation under certain unidentified conditions.

**Fig. 1:**
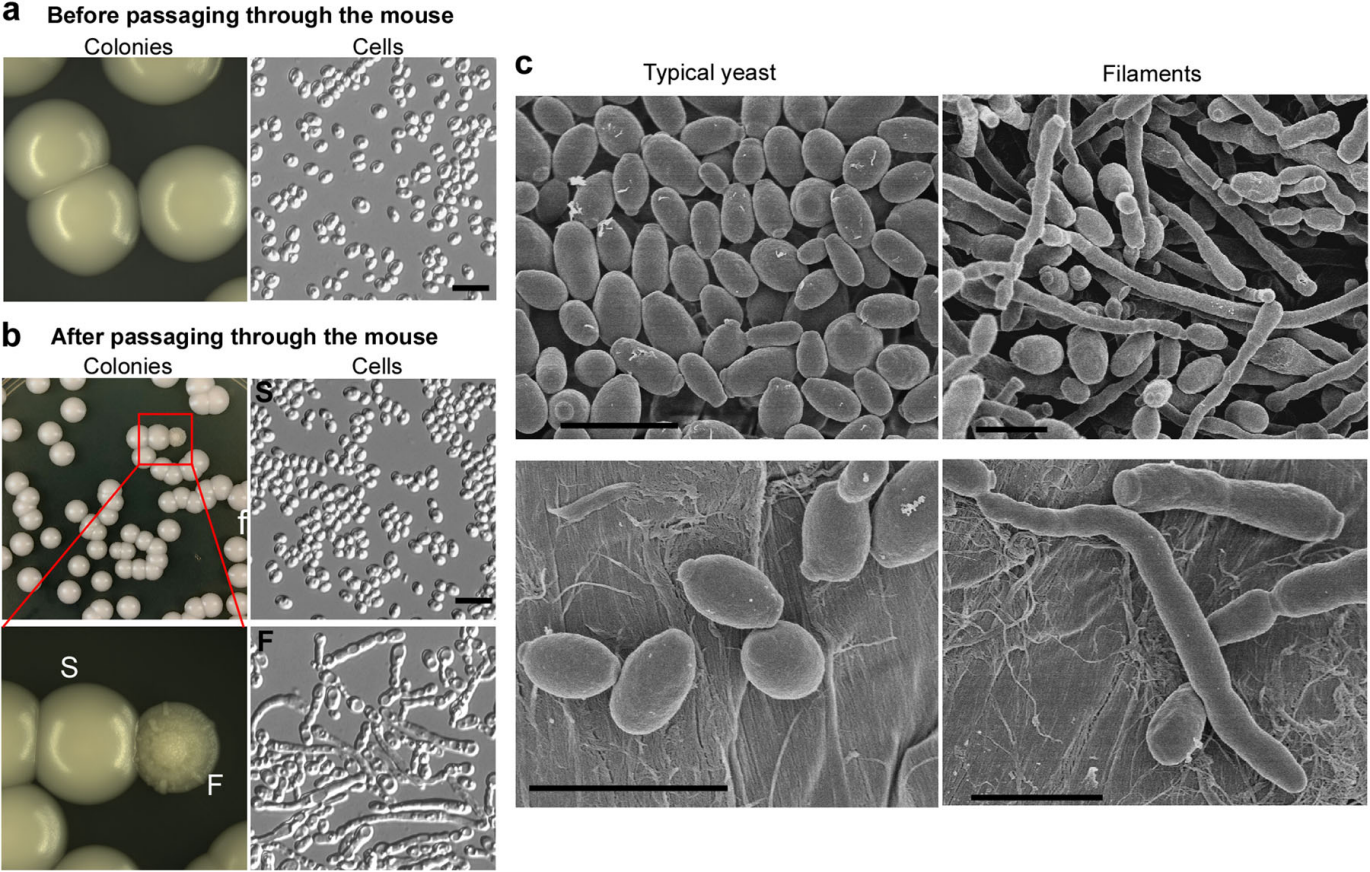
Observed colony and cellular morphologies of *C. auris*. **a** No filamentous colonies observed before passaging the animal. *C. auris* cells were grown on YPD medium for 2 days at 30 °C and then switched to 25 °C for five additional days. No filamentous colonies were observed (switching frequency < 0.001%). **b** Passage through the animal induces the filamentous morphology. *C. auris* cells were injected into mouse tail veins; cells were recovered from liver, kidney, brain, lung, and spleen tissues 24 h postinfection and replated on YPD medium. After 2 days of incubation at 30 °C, the cultures were then transferred to 25 °C for five additional days of incubation. Colonies shown were recovered from the liver and characterized as: smooth/typical yeast (S) or filamentous (F). Switching frequencies of typical yeast to filamentous colonies were (0.1 ± 0.2)% in the liver (colonies shown in the figure) and (0.4 ± 0.2)% in the kidney, respectively (totally, about 4000 colonies were analyzed). Scale bar represents 10 μm. **c** Scanning electron microscope (SEM) images of typical yeast and filamentous *C. auris* cells. *C. auris* cells were grown on YPD medium at 37 °C for 3 days and then switched at 25°C for three additional days of incubation. Scale bar represents 5 μm

When *C. auris* cells of the “typical yeast” form were cultured on YPD medium, they exclusively grew as the round yeast form (before mammalian passage, Fig. 1a). The “typical yeast” designation indicates that the yeast cells are unable to undergo filamentation upon environmental changes in vitro. However, when *C. auris* cells were recovered from the kidney and liver tissues of systemically infected mice 24 h postinfection and subsequently cultured on YPD medium, several highly wrinkled colonies containing very elongated filaments were observed (Fig. 1b). Interestingly, most of the filamentous colonies (~60%) were recovered from liver tissue, while a minor portion (~40%) came from kidney tissue. No filamentous cells were observed among cells recovered from the brain, lung, or spleen. The typical yeast-to-filament switching frequency in the liver was (0.1 ± 0.2)%, 100-fold higher than in the control cultures (< 0.001%) that were not passaged through a mammal. The frequency represents the ratio of filamentous colonies to normal yeast colonies after plating and growing on YPD plates.

**Fig. 2:**
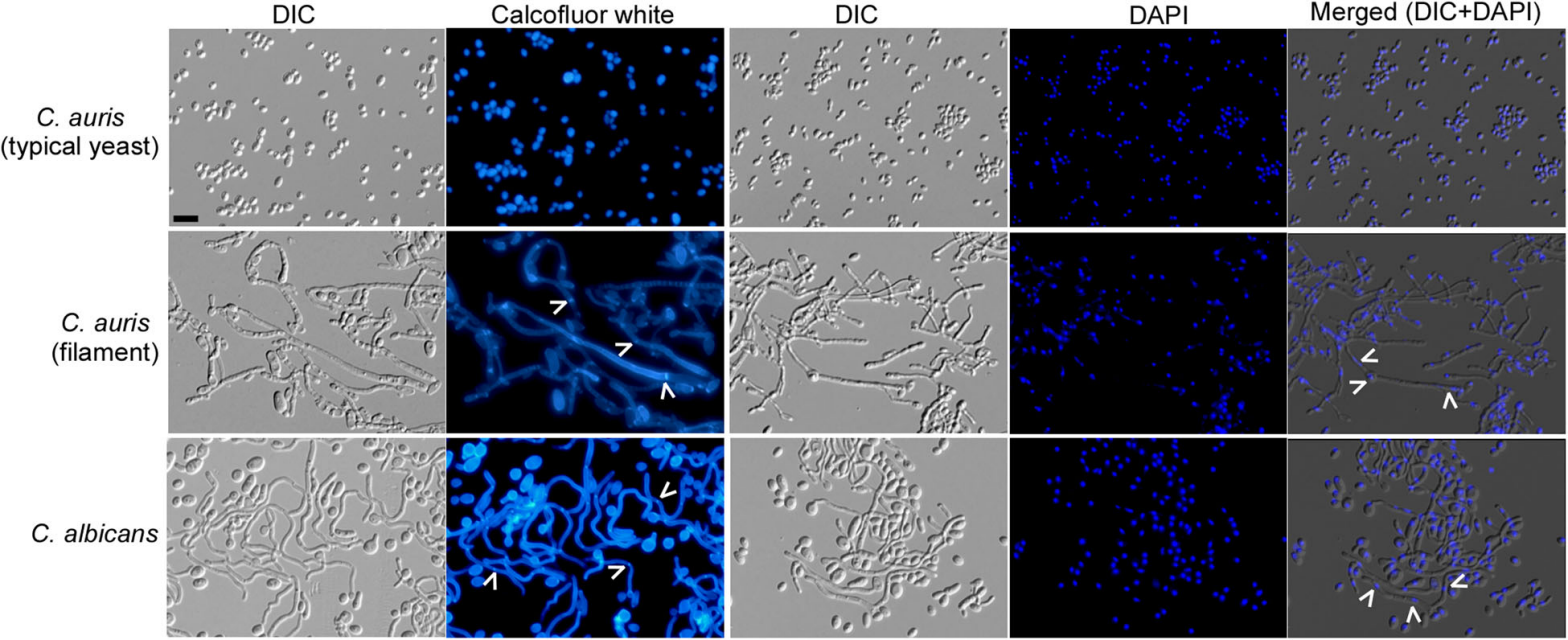
Calcofluor White and DAPI staining of *C. auris* and *C. albicans* cells. *C. auris* cells were grown on YPD medium at 37 °C for 3 days and then transferred at 25 °C for three additional days of incubation. *C. albicans* cells were grown in liquid YPD plus 10% serum medium at 37 °C for 6 h and collected for staining assays. White arrows indicate septin rings and nuclei. Scale bar represents 10 μm

*C. auris* cells exhibiting the filamentous phenotype were morphologically similar to the true hyphae of *C. albicans*, as revealed by scanning electron microscopy (SEM) and DIC microscopy (Figs. 1c, 2, and S1). Calcofluor White staining demonstrated that the filamentous *C*. *auris* cells have chitin rings (septa), and DAPI staining indicated that the filaments are often multicellular. A *C*. *auris* filamentous cell contained 3 to 23 vacuoles. The percentages of *C. auris* filamentous cells containing < 5, 5–9, or > 9 vacuoles were (31.7 ± 7.6)%, (47.2 ± 7.5)%, or (21.1 ± 8.4)%, respectively. In *C. albicans*, a filamentous cell contained 1 to 3 vacuoles. The percentages of filamentous cells containing one, two, or three vacuoles were (62.2 ± 8.2)%, (17.4 ± 4.4)%, or (20.5 ± 3.9)%, respectively (Fig. S1). Although the observed *C. auris* filaments generally resembled the morphology of *C. albicans* true hyphae, there were indeed some differences between the two cell types in terms of function and morphological characteristics. Therefore, the word “filaments”, rather than “hyphae”, is used in this study to describe the elongated *C. auris* cells.

### Effects of culture time and temperature on the maintenance of the filamentous phenotype

To test the effects of incubation time and temperature on the stability of the filamentous morphology, we grew *C. auris* cells on YPD plates at 25 or 37 °C for 3 days and then transferred half of the samples to an alternative temperature (from 25 to 37 °C or from 37 to 25 °C) for three additional days of growth (Fig. S2). Under all culture conditions, the cells with the typical yeast phenotype always maintained their yeast morphology (Fig. S2a). However, over 80% of filamentous cells converted to the yeast form after 3 days of incubation at either 25 °C or 37 °C (Fig. S2b). After further incubation at a constant or an alternative temperature for three additional days, the ratios of filamentous cells were significantly increased (Fig. S2b), suggesting that a long incubation period favors the growth of filaments, especially at 25 °C.

**Fig. 3:**
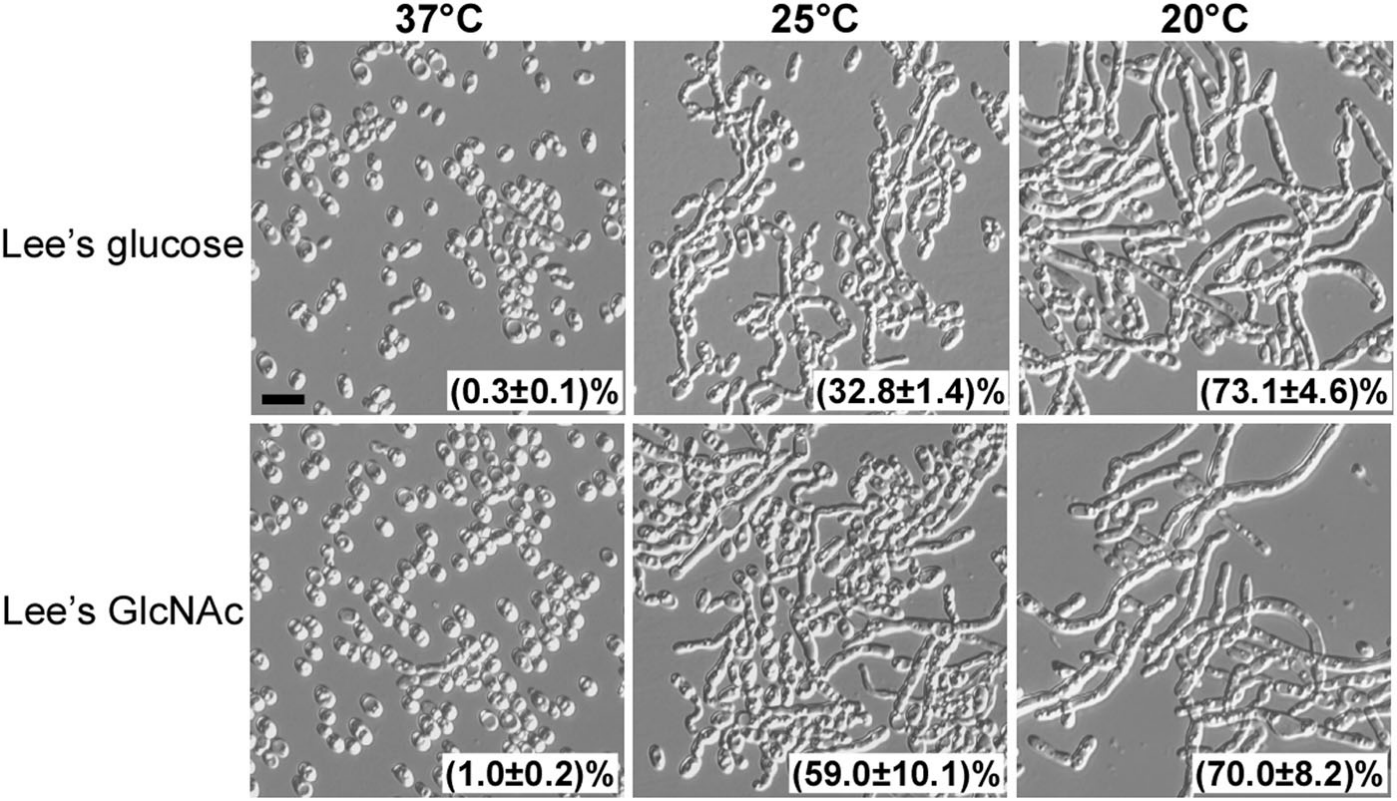
Effect of culture temperature on the persistence of the filamentous phenotype. Filamentous cells were plated on Lee’s glucose and Lee’s GlcNAc media for 7 days of growth at 37, 25, or 20 °C. Cellular morphology of representative colonies was examined. Percentage of filamentous cells are indicated. Approximately 1500–2000 cells were examined for each culture. Scale bar represents 10 μm

When filamentous cells were grown on synthetic media (Lee’s glucose or Lee’s GlcNAc), the inhibitory effect of high temperature on filamentous development was more obvious (Fig. 3). On synthetic media at 37 °C, all of the colonies became smooth (not shown) and more than 99% of the filamentous cells from representative colonies switched to the yeast form. However, at 20 °C, all of the colonies were wrinkled (not shown) and more than 70% of cells maintained a filamentous form. An intermediate level of filament-to-yeast switching frequency was observed at 25 °C (Fig. 3). Therefore, in contrast to the inducing effect of high temperatures on filamentation in *C. albicans*,  elevated temperatures promote filament-to-yeast switching in *C. auris*^10^.

### Filamentation-competent (FC) phenotype

As described earlier, high temperatures promoted a filament-to-yeast switch in *C. auris*. The yeast cells formed after this switch were morphologically indistinguishable from the original typical yeast cells (Figs. 1a and S2a). Typical yeast cells were unable to switch to the filamentous form under all of the tested in vitro culture conditions (Fig. S2a). However, yeast cells originating from filamentous cells, hereafter referred to as “filamentation-competent” or “FC yeast”, could undergo robust filamentation when grown at low temperatures (< 25 °C) (Figs. S2b and 3). The phenotype expressed by the FC yeast and the filamentous cell types is temperature-dependent, and the capability for filamentation is heritably maintained.

In liquid YPD medium, both FC yeast and filamentous cells grew as a mixture that contained > 85% of FC yeast and < 15% of filamentous cells, even at 25 °C (Fig. S3), suggesting that neither phenotype is stable in liquid cultures.

### Cellular memory of filamentation can be restored

**Fig. 4:**
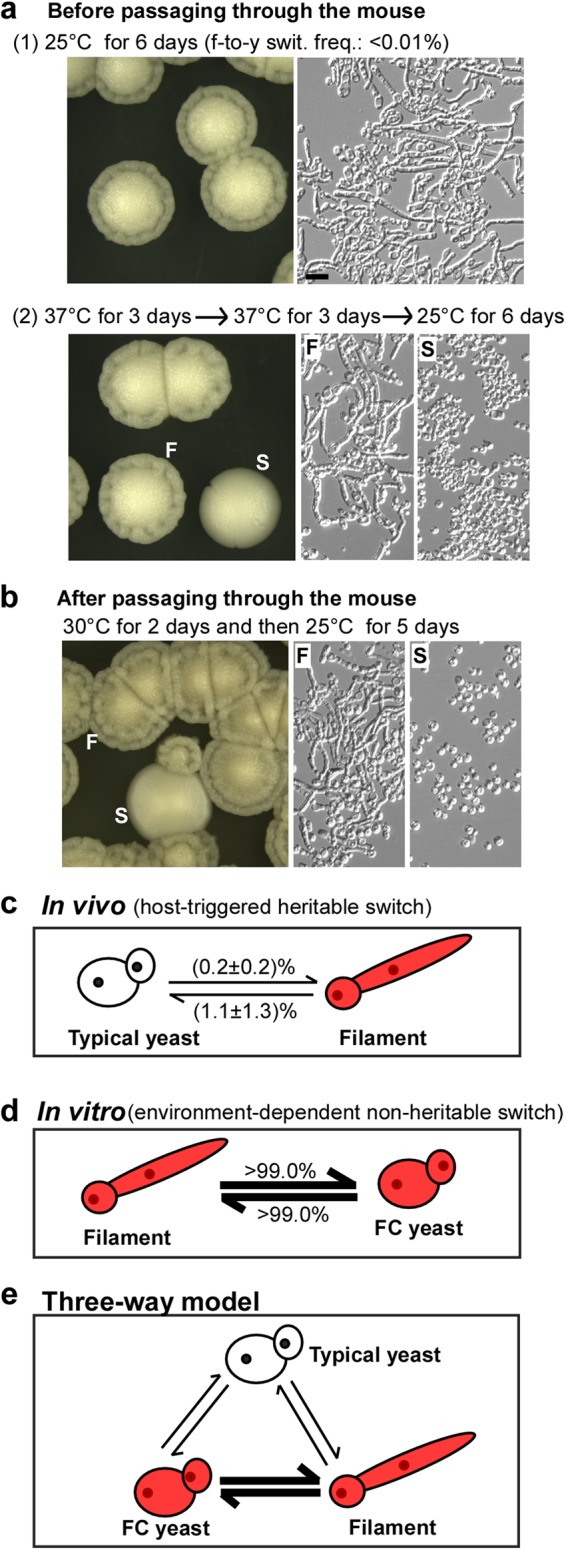
In vivo and in vitro loss of filamentation phenotype observations in *C. auris*. **a** Morphologies without passage through the mouse: (1) filamentous cells were plated on YPD medium for 6 days of growth at 25 °C (no switching to the typical yeast colonies were observed, f-to-y swit. frequency < 0.01%); (2) filamentous cells were grown on YPD medium at 37 °C for 3 days, replated, and grown at 37 °C for three additional days before being replated and grown at 25 °C for 6 days (switching frequency from filamentous-to-typical yeast colonies was 1.2 ± 0.2%). **b** Morphologies after passage through the mouse. Filamentous cells were injected into the mouse via the tail vein. Cells recovered from tissues were grown on YPD medium at 30 °C for 2 days and then transferred at 25 °C for 5 days of incubation. Colonies shown were recovered from kidney tissues and classified as smooth (S, typical yeast) or filamentous (F). **c** In vivo model. Typical yeast-filament switching frequencies represent the average percentages of colonies with an alternative phenotype after passage through the animal and replating and culturing on YPD medium at 25 °C. These host-triggered switches are rare and heritable. **d** In vitro FC yeast-filament switches. FC yeast designation indicates a filamentation-competent form of *C. auris*, an intermediate cell type capable of returning from a morphologically yeast phenotype to a filamentous form following exposure to 25 °C growth conditions. The frequency of the FC yeast-to-filament switch is higher than 99% at 25 °C and the frequency of the filament-to-FC yeast switch is higher 99% at 37 °C (~1000 colonies were analyzed for each switch). Therefore, the morphological switches between the FC yeast and filamentous forms are temperature-dependent and nonheritable. **e** The typical yeast, FC yeast, and filamentous cell types form a three-way switching system that combines a heritable (typical yeast-to-FC yeast/filamentous) and a nonheritable (FC yeast-to-filamentous) switch. Thin and thick lines indicate low and high switching frequencies, respectively

Next, the capability of filamentous cells to switch back to the typical yeast form (which is unable to undergo filamentation under in vitro conditions) was examined. At 25 °C, filamentous cells could only form wrinkled colonies, with a filament-to-typical yeast switching frequency of < 0.01% (Fig. 4a). Filamentous cells were then plated on YPD medium and incubated at the host physiological temperature (37 °C) for 3 days. After two passages at 37 °C, cells from representative colonies were replated on YPD medium and incubated at 25 °C for 6 days; most of the colonies were observed to be wrinkled, while (1.2 ± 0.2)% of the colonies were smooth and consisted of yeast cells (Fig. 4a). Taken together, these data suggest that two passages at 37 °C leads to the loss of the FC phenotype in a small number of cells.

Next, systemic mouse infections were performed by injecting filamentous *C. auris* cells into the tail vein (Fig. 4b). Fungal cells recovered from different animal tissues were plated on YPD medium and incubated at 30 °C for 2 days followed by transfer to 25 °C for another 5 days of incubation. We observed that (1.1 ± 1.3)% of the colonies formed by fungal cells recovered from mammalian tissues were smooth and consisted of typical yeast cells (Fig. 4b). Our results suggest that passage through a mammalian body can potentially erase the cellular memory of filamentation and promote the switch to a typical yeast phenotype.

Taken together, our results reveal that the ability to switch between the typical yeast and filamentous forms is heritable and triggered by passage through the animal (Fig. 4c), whereas the switch between FC yeast and the filamentous form is nonheritable and temperature-dependent (Fig. 4d). The three cell types form a novel switching system that combines a heritable transition (between typical yeast and filamentous/FC yeast) and a nonheritable transition (between FC yeast and filamentous) (Fig. 4e).

### Global gene expression profiles of *C. auris* typical yeast and filamentous cells

**Fig. 5:**
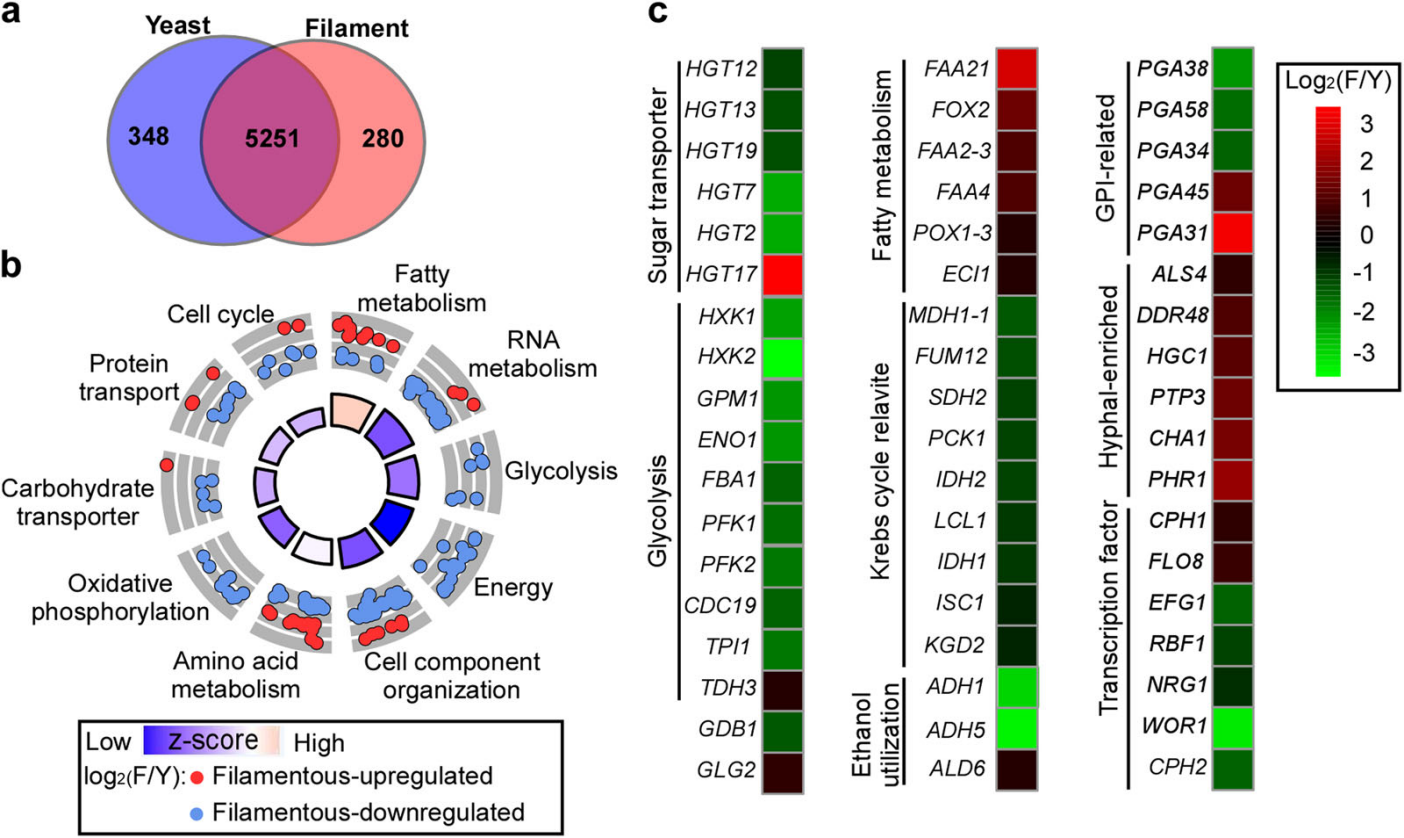
Comparison of global gene expression profiles of *C. auris* in typical yeast and filamentous cells. **a** Venn diagram depicting differentially expressed genes. A twofold difference cut off and false discovery rates (FDRs) less than 0.05 were used to define differentially expressed genes. **b** GO enrichment analysis of differentially expressed genes was conducted. Red or blue circles represent upregulated or downregulated genes in filamentous cells, respectively. The inner cycle bars represent statistical significance. **c** R package heatmap was used to depict selected differentially expressed genes. Functional categories of genes are indicated. Log_2_(F/Y), Log_2_ (read counts of filamentous cells/read counts of yeast cells)

To systemically reveal the differences between *C. auris* typical yeast and filamentous cells, an RNA-Seq analysis was performed. The FC cell type was not examined because FC cells are unstable and undergo filamentation under the culture condition used. In total, 628 genes with greater than twofold differences in their expression levels between the two cell types were identified (Fig. 5a). Among the identified genes, 348 were upregulated in typical yeast cells, and 280 were upregulated in filamentous cells. Differentially expressed genes drive a diverse range of cellular functions, including metabolism, transcriptional regulation, oxidative phosphorylation, cell cycle control, and cell component organization (Fig. 5b and Dataset S1).

A large subset of metabolism-associated genes was differentially expressed between the typical yeast and filamentous cell types. Genes belonging to both the glycolytic and Krebs cycle pathways were upregulated in typical yeast cells, whereas fatty acid (β-oxidation) metabolism-related genes were enriched in filamentous cells (Fig. 5c and Dataset S1). For example, *HXK1* (encoding the hexokinase isoenzyme), *PFK1* and *PFK2* (encoding two subunits of heterooctameric phosphofructokinase), and *CDC19* (encoding the pyruvate kinase) were expressed at higher levels in typical yeast cells than in filamentous cells. With the exception of *HGT17* (encoding an MFS family glucose transporter), other sugar transporter-encoding genes, including *HGT2, HGT7, HGT12, HGT13*, and *HGT19*, were upregulated in typical yeast cells. Consistently, *TYE7*, which encodes a transcription factor required for the control of glycolysis, was comparatively upregulated in typical yeast cells.

The genes encoding the homologs of the *C. albicans* hyphal regulators were upregulated in filamentous cells, suggesting some similarities in filamentation between *C. auris* and *C. albicans*. For example, *HGC1* and *ALS4*, which encode a G1 cyclin-related protein and a GPI-anchored protein, respectively, were upregulated in filamentous cells. A number of conserved transcriptional regulator-encoding genes that govern filamentous growth in *C. albicans* were also differentially expressed between the typical yeast and filamentous *C. auris* cells. For example, *CPH1* and *FLO8* were comparatively upregulated in filamentous cells, while *NRG1, CUP9*, and *RBF1* had higher expression levels in typical yeast cells. *NRG1* and *CUP9*, which encode conserved general transcriptional repressors in fungi, often play inhibitory roles in the regulation of filamentous growth^27^. *EFG1* and *WOR1* encode a negative and a positive regulator of white-to-opaque switching in *C. albicans*, respectively^28^. *EFG1* is also known to be required for filamentous growth in *C. albicans*^29^. Interestingly, both *EFG1* and *WOR1* were downregulated in filamentous *C. auris* cells. The *C. auris* homolog of *OP4*, an opaque-specific gene in *C. albicans*, exhibited a higher transcriptional level in filamentous cells than in typical yeast cells.

A subset of cell wall-associated and GPI-anchored protein-encoding genes was differentially expressed between the two cell types (Fig. 5c). Typical yeast cells demonstrated elevated expression levels of cell wall-related genes, including *PGA34, PGA38*, and *PGA58*, and *PGA31* and *PGA45* were upregulated in filamentous cells. Many genes encoding histone proteins (*HHO1, HHT2, HHF22, HTA2*, and *HTA3*) or histone modifiers (*PHO15, PIS523355.1*, and *PIS55449.1*) were downregulated in filamentous cells, implying that epigenetic regulation may be involved in the heritable typical yeast-filament switch phenotype. Iron metabolism-associated genes (*SFU1, FRE8, FTR1*, and *FTR2*) were upregulated in filamentous cells. To verify the RNA-Seq data, we selected 17 identified differentially expressed genes and performed quantitative RT-PCR assays. As shown in Fig. S4, the expression patterns of these genes were generally consistent with those of the RNA-Seq assays. Taken together, our results indicate that the typical yeast and filamentous cell types exhibit highly differential gene expression profiles in *C. auris*.

### Differential SAP activities

**Fig. 6:**
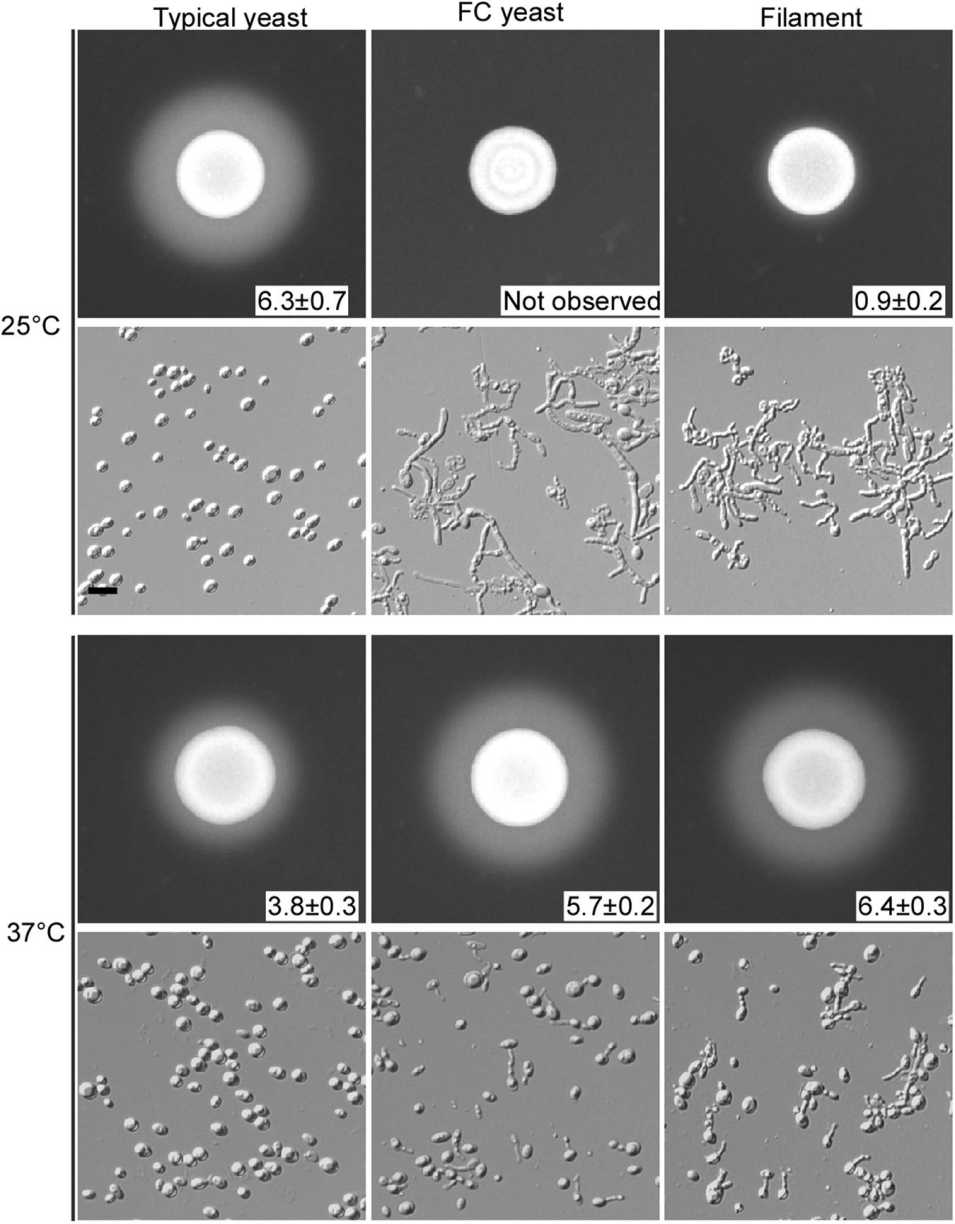
Secreted aspartyl proteinase (SAP) activity of typical yeast, FC yeast, and filamentous *C. auris* cells. Cells were spotted on YCB-BSA medium (5 × 10^6^ cells) and grown at 25 or 37 °C for 5 days. The width of the white precipitation zone (mm) is reported. Cellular morphologies after 5 days of growth are also shown. Scale bar represents 10 μm

Secreted aspartic proteinases (SAPs) are a key virulence factor in pathogenic fungi^30^. Although most SAP-encoding genes showed comparable transcription levels in typical yeast and filamentous *C. auris* cells, two *SAP* genes (typical yeast-enriched PIS52298.1 and filamentous-enriched PIS48635.1) were differentially expressed (Dataset S1). Posttranscriptional regulation may also affect SAP activity in both cell types. Therefore, SAP activity was tested in typical yeast, FC yeast, and filamentous cells using the YCB-BSA assay^26^. As shown in Fig. 6, typical yeast cells exhibited higher SAP activity at 25 °C than at 37 °C, whereas FC yeast and filamentous cells showed higher SAP activity at 37 °C than at 25 °C, as indicated by the size of the BSA precipitation zones. Of note, although the original morphologies of the FC yeast and filamentous cells were distinct, the two cell types exhibited similar morphologies after 5 days of growth at both 25 and 37 °C. Our results suggest both environmental temperature and morphological transitions regulate SAP activity in *C. auris*.

### Virulence of typical yeast and filamentous *C. auris* cells

**Fig. 7:**
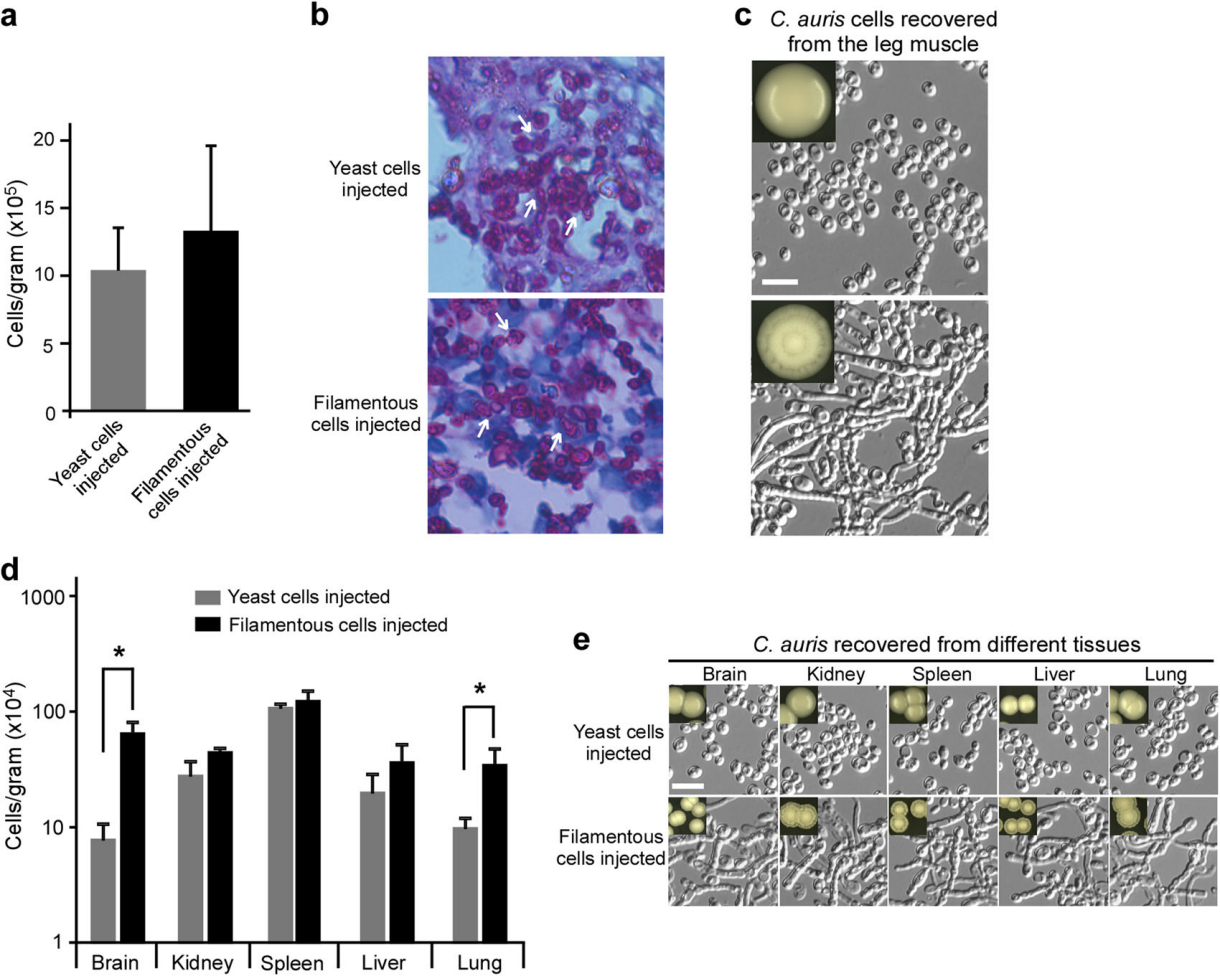
Virulence of typical yeast and filamentous *C. auris* cells in a mouse model of fungal burden. **a** Fungal burdens were determined using excised leg muscle samples. Mice were injected with typical yeast or filamentous cells intraperitoneally. After 24 h of infection, mice were euthanized humanely. **b** Periodic acid–schiff (PAS) stained sections of spleens from infected animals were imaged. Tissues from intraperitoneally injected mice were fixed, sectioned, and stained for microscopy assays. **c** Morphological analysis of *C. auris* cells recovered from leg tissues described in panel (**a**). **d** Fungal burdens as determined through in vivo systemic infection mouse model. Typical yeast or filamentous cells were injected via the mouse tail vein. Fungal burdens of multiple organs were analyzed following 24 h of infection. **e** Morphological analysis of *C. auris* cells recovered from organ tissue samples described in panel (**d**). Recovered cells were plated and grown on YPD medium at 30 °C for 2 days and then transferred at 25 °C for 5 days of incubation. Scale bar represents 10 μm

To compare the tissue-invading capability of typical yeast and filamentous *C. auris* cells, intraperitoneal infection assays were performed, and the fungal burden in the leg muscles was assessed. No significant difference in fungal burden was observed between typical yeast and filamentous cells (Fig. 7a). Observation of sectioned and stained tissues revealed that typical yeast cells maintained their original phenotype. However, most of the filamentous cells morphologically transitioned to the FC yeast phenotype (Fig. 7b). After recovery from infected leg tissues, FC yeast cells returned to their original filamentous phenotype after plating on YPD medium and incubation at 25 °C (Fig. 7c).

A fungal burden assay using a systemic mouse infection model was then performed. As shown in Fig. 7d, the fungal burdens of typical yeast and filamentous cells in the kidney, liver, and spleen were comparable. However, the fungal burdens of filamentous cells in the brain and lung were significantly higher (*p* < 0.05) than those of typical yeast cells. Similar to the results observed during the intraperitoneal infection assays, fungal cells recovered from different tissues displayed their original phenotypes when plated on YPD medium and incubated at 25 °C (Fig. 7e). These results indicate that both typical yeast and filamentous cells can return to their original phenotypes after passaging through a mammal, although filamentous cells shift to the FC form during infection.

**Fig. 8:**
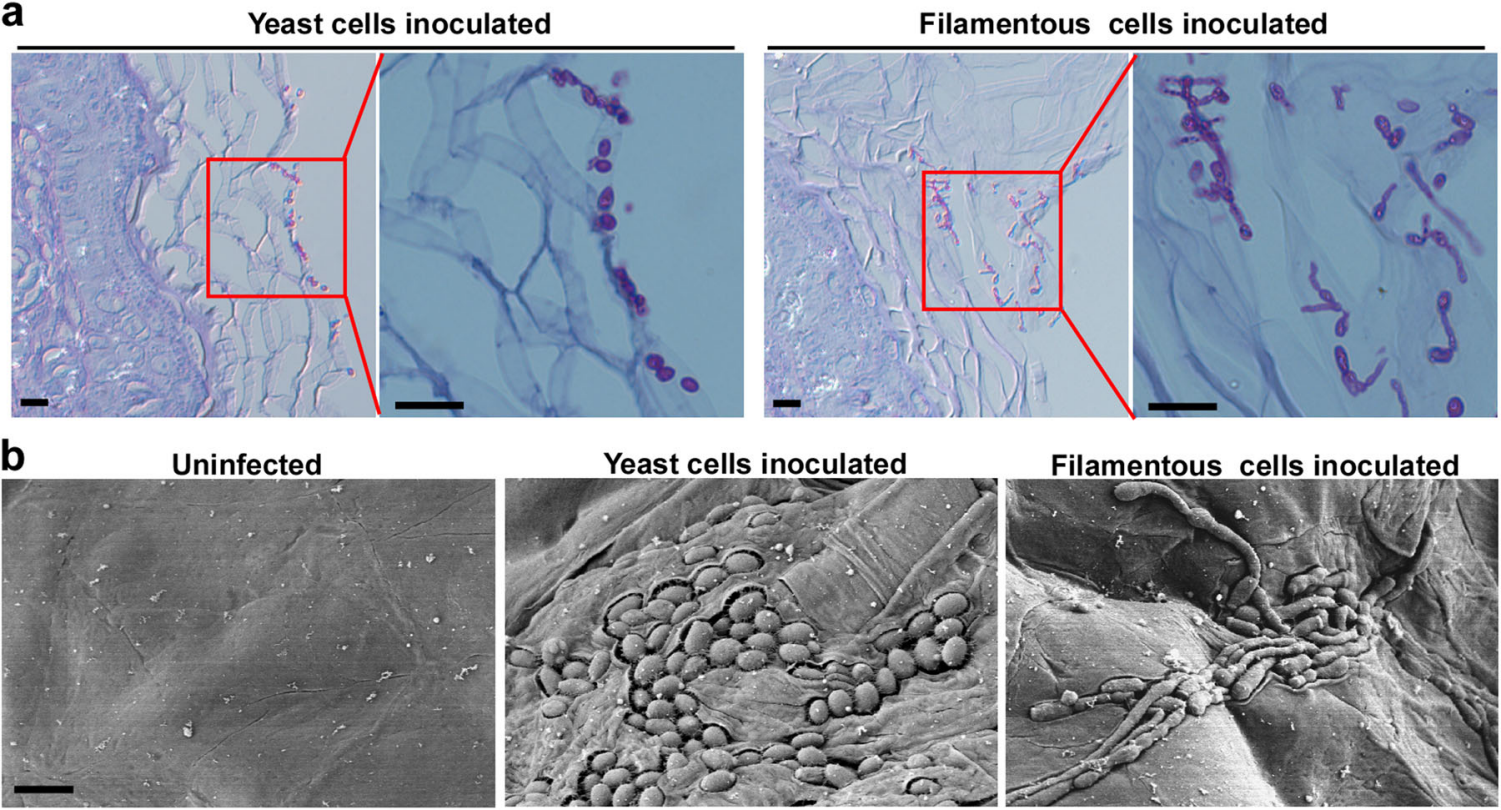
In vivo assessment of typical yeast and filamentous *C. auris* cell virulence in a mouse skin infection model. **a** 1 × 10^7^ typical yeast or filamentous cells were inoculated onto the skin of 2–5-day-old mice. After 24 h of infection, mice were humanely euthanized. Histopathological PAS sections of infected skin tissues were used for microscopy assays. Scale bar represents 10 μm. **b** Scanning electron microscope (SEM) images of infected skin tissues. 4 × 10^6^ typical yeast or filamentous cells were inoculated onto the skin of 2–5-day-old mice. After 24 h of infection, mice were euthanized humanely. Infected skin tissues were used for SEM assays. Scale bar represents 5 μm

Unlike *C. albicans* and other pathogenic *Candida* species, which are commensals of the human gut, *C. auris* may be a commensal of the skin^18,24^. In the mouse skin infection model, typical yeast cells were found to predominately colonize at the skin’s surface, whereas filamentous cells often invaded the epidermal layer while maintaining their phenotype (Fig. 8a, b). These results suggest that filamentous cells are more stable during skin infections than during systemic and intraperitoneal infections.

## Discussion

A striking feature of pathogenic *Candida* species is phenotypic plasticity. In this study, we report the discovery of filamentation and a novel phenotypic switching system in the recently evolved fungal pathogen *C. auris*. This switching system involves three distinct cell types: typical yeast, FC yeast, and a filamentous form (Fig. 4c–e). All of the cell phenotypes are heritable in terms of filamentation potential. Typical yeast cells are filamentation-incompetent, whereas FC yeast and filamentous cells are filamentation-competent under specific in vitro culture conditions. *C. auris* FC yeast cells are morphologically similar to typical yeast cells, although they are functionally comparable to filamentous cells in terms of SAP secretion and filamentation. The bistable switch between the typical yeast and FC yeast/filamentous cell types is similar to the white-opaque transition in *C. albicans*^11^. Furthermore, the nonheritable switch between the FC yeast and filamentous phenotypes is similar to the highly environmentally dependent yeast-filament transition observed in *C. albicans*^10^. Passage through tissues of a mammalian host promotes the bistable typical yeast-FC yeast/filamentous switch (Fig. 4c–e). Once cells have committed to an alternative phenotype, they can maintain and transmit that phenotype to future generations.

Global transcriptional profile analysis reveals a large set of genes that are differentially expressed between the typical yeast and filamentous cell types (Fig. 5 and Dataset S1). As expected, a number of filamentation-specific genes and *C. albicans* regulatory homologs were observed to be upregulated in filamentous *C. auris* cells. The exceptions to this observation were *EFG1* and *CPH2*, positive regulators in *C. albicans* that were downregulated in filamentous *C. auris* cells. These data suggest that the two species have both conserved and unique filamentation regulators.

Differential expression of cell wall or cell surface-related genes was also identified, implicating that changes in cell surface antigens are associated with the capability to undergo filamentation. Antigenic variation is a common strategy employed by microbial pathogens to escape host immune attack^1,3^. The most striking difference between the typical yeast and filamentous cell types was variability among the expression levels of metabolism genes. In filamentous *C. auris* cells, both Krebs cycle- and glycolytic pathway-associated genes were downregulated, while fatty acid metabolism-related genes were upregulated. Metabolic specialization in pathogenic microbes is often associated with commensalism, pathogenesis, and antigenic variation^1,31^. Distinct metabolic modes could benefit specific cell types in survival and persistence within diverse host niches. One would expect *C. auris* to use the same strategies to adapt to its host to reap the overall benefits of its commensal and pathogenic lifestyle.

Although both the typical yeast-FC yeast/filamentous switch in *C. auris* and white-gray-opaque transition in *C. albicans* are heritable and involved in virulence regulation^32^, there are distinct features between the two morphological switching systems. First, three cell types (typical yeast, FC yeast, and filamentous) have been identified as components of the *C. auris* switching system, and three cell types (white, gray, and opaque) have been identified in the white-gray-opaque switching system of *C. albicans*. Although the switch between the typical yeast and filamentous/FC yeast phenotypes is heritable, the transition between FC yeast and the filamentous form in *C. auris* maintains an environmental dependence. The latter switch is similar to the yeast-filament switching documented in *C. albicans*. Incubation at the host physiological temperature was demonstrated to reverse the induction of the filamentation phenotypes in the two *Candida* species. However, all of the transitions between the white and gray, white and opaque, and gray and opaque phenotypes are heritable^32^. Recently, another heritable phenotype, namely, the GUT cell type, has also been reported in *C. albicans*^33^. The GUT morphology is induced upon passage through the mammalian gut and represents a commensal phenotype of *C. albicans*.

Second, the global transcription profiles of typical yeast and filamentous *C. auris* cells exhibit unique features. For example, *C. auris* typical yeast cells show increased expression levels of genes involved in the Krebs cycle and genes associated with glycolysis. In *C. albicans*, upregulation of glycolysis-related genes in white cells and Krebs cycle genes in opaque cells were observed^1^.

Third, the expression patterns of SAP virulence factors in *C. auris* differ from the two switching systems in *C. albicans*. However, it remains to be investigated whether or not the *C. auris* switch is associated with sexual mating, as has been documented for white-opaque switching in *C. albicans*^34^. Together, the documented similarities between the mechanisms of environmentally triggered phenotypic switches are adequate to suggest a shift towards increased virulence.

Pathogenic *Candida* species have been thought to be common members of the human microbiota. Data have indicated that *C. auris*, as a skin commensal, is unlikely to be found in the human gut or mouth, common locations of *C. albicans* and *C. tropicalis* colonization^18,24^. These distinct ecological niches may shape individual species-specific responses to environmental changes. Systemic infection assays demonstrate that the typical yeast and filamentous cell types exhibited comparable virulence and fungal burden in liver, kidney, and spleen tissues. However, the fungal burdens in brain and lung tissues were much higher in cells displaying the filamentous or FC yeast phenotypes compared with typical yeast cells. When observed using a skin infection model, filamentous cells were more invasive than typical yeast cells (Fig. 8). The skin temperatures of mammals are relatively low, making skin colonization conducive to expression of the filamentous phenotype by *C. auris*. Furthermore, filamentous *C. auris* cells secrete less SAP than typical yeast cells at low temperatures, potentially further facilitating a commensal lifestyle on the skin (Fig. 6). Our data demonstrate that the distinct cell phenotypes of *C. auris* exhibit different virulence levels in a mammalian host.

Taken together, the typical yeast-FC yeast/filamentous switch in *C. auris* represents a novel phenotypic system in fungi. This system couples a heritable switch (the typical yeast-FC yeast/filament transition) with the environmentally dependent FC yeast-filament transition. The described switching system likely plays roles in virulence and metabolism; however, more intensive investigation is required to fully elucidate its full implications in an evolving *C. auris*.

## Materials and methods

### Strains and culture conditions

YPD (10 g/L yeast extract, 20 g/L peptone, and 20 g/L glucose) was used for routine growth of *Candida auris* and *Candida albicans*. The yeast extract for the YPD medium was bought from the Angel Company (Hubei, China). Peptone was from Oxoid Ltd. Company (Hants, UK) and glucose was from Beijing Chemical Works (Beijing, China). The *C. auris* strain BJCA001 and the *C. albicans* strain SC5314 were used for this study^26,35^.

### Morphological switching assays

To examine typical yeast-filament switching in vivo, *C. auris* cells were injected and subsequently recovered from mice. Cells were then plated and passaged on YPD medium at 30 °C for 2 days and subsequently transferred to 25 °C for five additional days of incubation. Colony and cellular morphologies were recorded.

For the in vitro switching assays, typical yeast or filamentous cells were plated onto YPD, Lee’s glucose, or Lee’s GlcNAc medium^14^. The culture conditions are described in the relevant figures and in the main text. The switching frequency was defined as the number of colonies with an alternative phenotype divided by the number of total colonies multiplied by 100 to give a percentage. Data were reported as switching frequency percentage ± standard deviation (SD).

### DAPI, Calcofluor White, and FM4-64 staining assays

Typical yeast or filamentous *C. auris* cells of were grown on YPD agar medium at 37 °C for 3 days and then transferred to 25 °C for three additional days of growth. *C. albicans* yeast cells were grown in liquid YPD medium at 37 °C for 6 h. For the induction of filaments, *C. albicans* cells were grown in liquid YPD plus 10% serum medium at 37 °C for 6 h. Cells were collected and washed with ddH_2_O. Differential interference contrast (DIC) optics were used for standard cellular morphology assays. 4′,6-diamidino-2-phenylindole (DAPI, Sigma-Aldrich, Inc., Beijing) was used for nuclear staining, and Calcofluor White (Sigma-Aldrich, Inc., Beijing) was used for septa/chitin staining as previously described^26,32^. The dye FM4-64 was used for vacuole staining as previously described^36^.

### Secreted aspartyl proteinase (SAP) activity assay

YCB-BSA medium was used for the SAP activity assays as previously reported^26^. Typical yeast or filamentous cells were grown on YPD medium at 37 °C for 3 days and then transferred to 25 °C for three additional days of growth. FC yeast cells were grown on YPD plates at 37 °C for 2 days. All cells were then collected and washed with ddH_2_O. Next, ~5 × 10^6^ cells in 5 μL ddH_2_O were spotted onto YCB-BSA plates and cultured at 25 °C or 37 °C for 5 days. The width of the BSA precipitation rings (halos) was recorded and used as a measurement of SAP activity. Three biological repeats were performed.

### Animal experiments

All animal experiments were performed according to the guidelines approved by the Animal Care and Use Committee of the Institute of Microbiology, Chinese Academy of Sciences. The mouse systemic infection experiments were performed as previously described, with minor modifications^26^. For the fungal burden assays, five-week-old female BALB/c mice (*n* = 4) were used for each infection condition. Typical yeast or filamentous cells (2 × 10^8^ cells) were suspended in 250 μL 1 x PBS and intraperitoneally injected into each mouse. Systemic infections were conducted by suspending 2 × 10^7^ cells in 250 μL 1 x PBS and intravenously injected into the mice. After 24 h of infection, the mice were humanely euthanized. The spleens from the intraperitoneally infected mice were used for the histopathology assays, while leg muscle tissue was used for the fungal burden assays. Brain, kidney, spleen, liver, and lung samples of intravenously infected mice were used for the fungal burden and in vitro morphological assays.

Newborn BALB/c mice (2–5 days old) were used for the skin infection assays as described previously^32^. For SEM assays, 4 × 10^6^ cells in 4 μL of ddH_2_O were spotted onto the back skin of a newborn mouse for each phenotype. For skin histopathology assays, 1 × 10^7^ cells of each type in 4 μL of ddH_2_O were spotted onto the back. After the water had evaporated, a small piece of sterile filter paper was placed over the infection site and fixed in place with first aid tape. After 24 h, the infected areas were excised for SEM or histopathological assays.

### Histopathological assay

Spleen or skin tissues of infected mice were fixed with 10% (weight/volume) buffered formalin, washed, dehydrated, and embedded in paraffin wax, as previously described^37^. The samples were sectioned and stained with periodic acid–Schiff (PAS) for microscopy assays.

### Scanning electron microscopy (SEM)

SEM assays were performed as described in our previous publications with slight modifications^26,32^. Typical yeast or filamentous cells were grown on YPD agar medium at 37 °C for 3 days and then transferred to 25 °C for three more days of incubation. Endpoint cells were used for SEM assays. Infected skin tissues were sectioned, fixed with 2.5% glutaraldehyde, and washed three times with 1 x PBS. The samples were then dehydrated using gradually increasing concentrations of ethanol (50, 70, 85, 95, and 100%), dried, and then coated with gold. The prepared samples were then imaged using a Quanta 200 Scanning Electron Microscope (Thermo Fisher Scientific, USA)).

### Quantitative real-time PCR (Q-RT-PCR) and RNA-Seq assays

Typical yeast or filamentous cells of *C. auris* were grown on YPD agar medium at 37 °C for 3 days and then transferred to 25 °C for three additional days of growth. This culture condition was found to be optimal for filamentous growth in *C. auris*. Cells were collected and total RNA extracted for the RNA-Seq and Q-RT-PCR analyses. One microgram of total RNA per sample was used to synthesize cDNA with RevertAid Reverse Transcriptase (Thermo Scientific, Inc.). Quantification of the transcripts was performed in a Bio-Rad CFX96 real-time PCR detection system using SYBR green. The expression levels of each experimental sample were normalized to that of *C. auris ACT1*.

For the RNA-Seq analysis, approximately 10 million (M) reads were obtained by sequencing each library. The library products were sequenced using an Illumina HiSeq 2000 instrument (Berry Genomics Co., Beijing). The raw data were filtered by removing adapter and low-quality reads using FastQC v0.11.5 (http://www.bioinformatics.babraham.ac.uk/projects/fastqc). Clean reads were aligned to the previously established *C. auris* assembly from NCBI (GCA_002759435.2) using HiSat2 v2.0.5. Transcript expression levels were estimated with Stringtie v1.3.3b^38^. Differentially expressed genes were analyzed using the DESeq2 package for R^39^. The GO enrichment analysis was performed based on the Gene Ontology Consortium terms (http://www.geneontology.org/). The heatmap and GOplot packages for R were used to visualize clustering^40^.

## Electronic supplementary material

Dataset S1

Figure S1

Figure S2

Figure S3

Figure S4

Supplementary information
