# Supplementary figures and images for: Filamentation in Candida auris, an emerging fungal pathogen of humans: passage through the mammalian body induces a heritable phenotypic switch
Source: Emerg Microbes Infect. 2018 Nov 28;7:188. doi: 10.1038/s41426-018-0187-x (PMC6258701; doi:10.1038/s41426-018-0187-x)

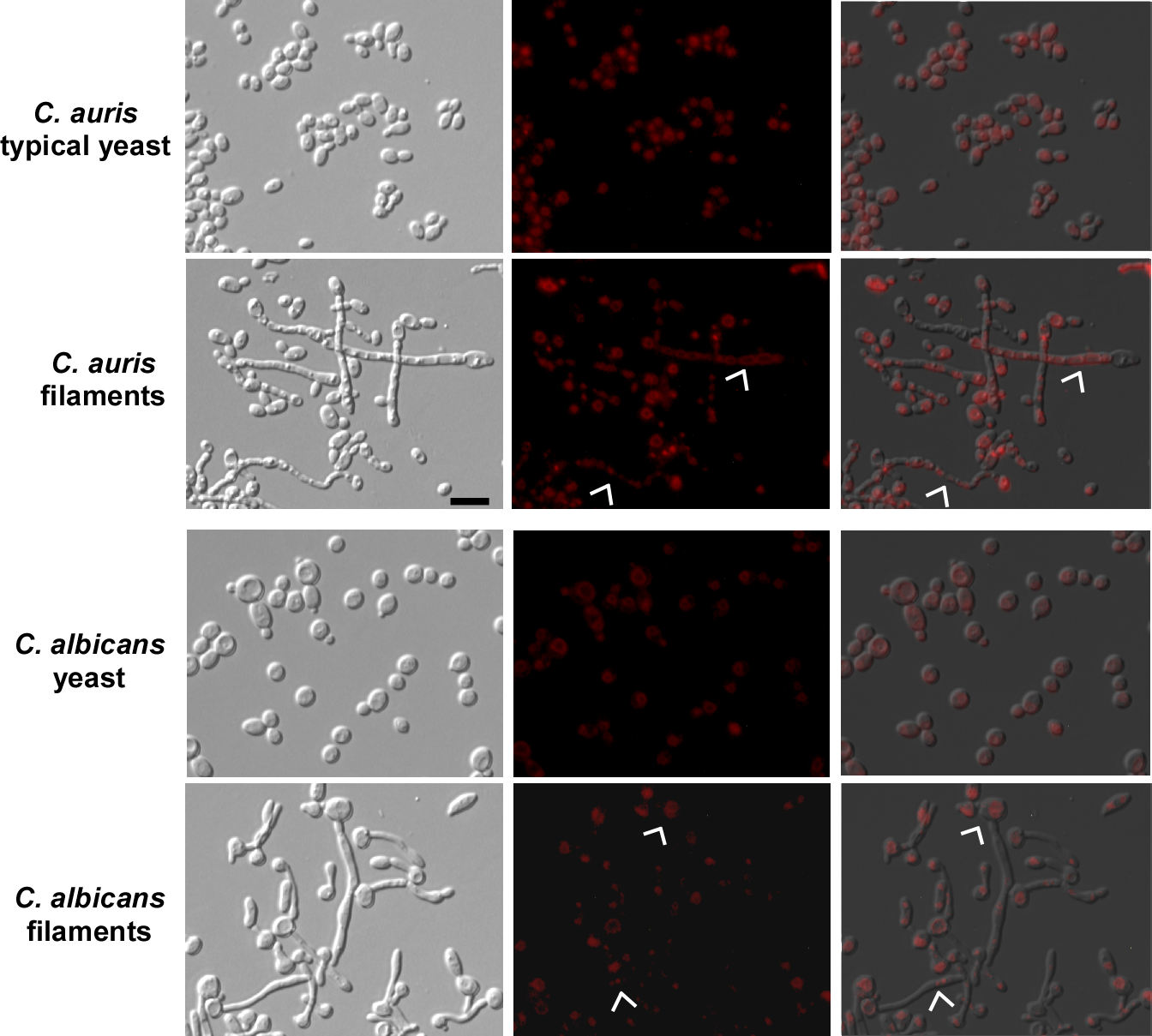

Supplement: Supplementary file 2 — Figure S1 [file 41426_2018_187_MOESM2_ESM.tif]

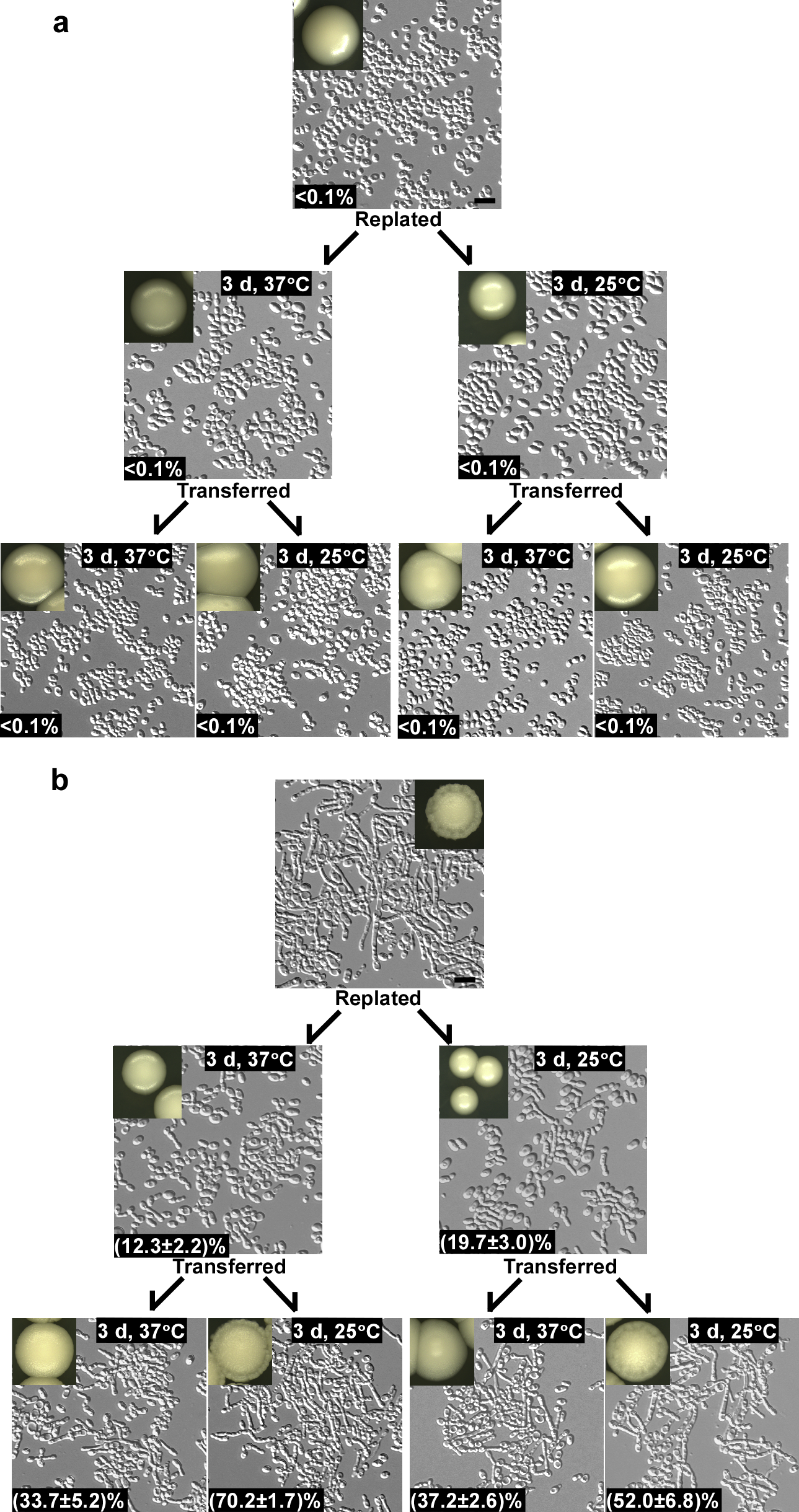

Supplement: Supplementary file 3 — Figure S2 [file 41426_2018_187_MOESM3_ESM.tif]

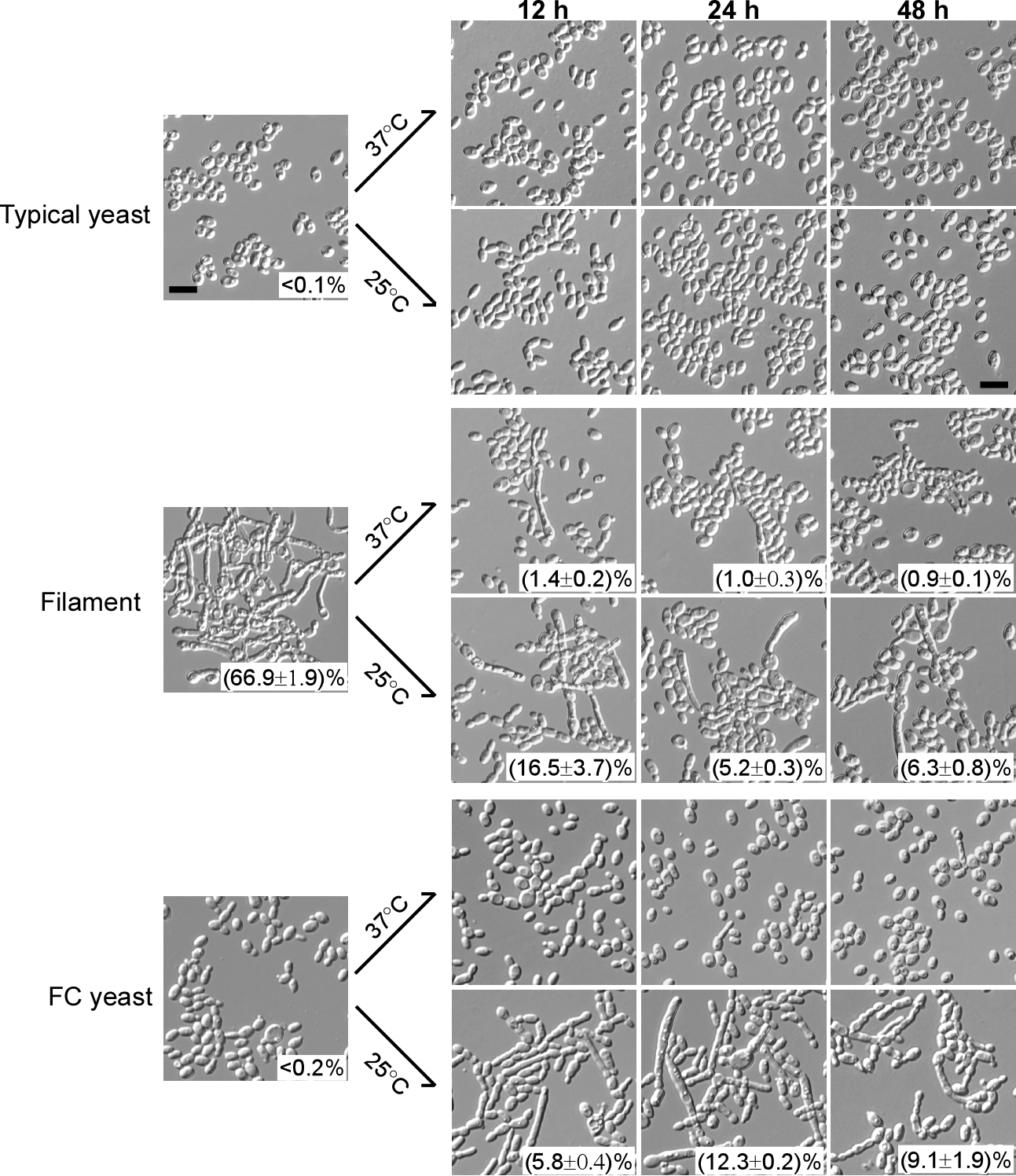

Supplement: Supplementary file 4 — Figure S3 [file 41426_2018_187_MOESM4_ESM.tif]

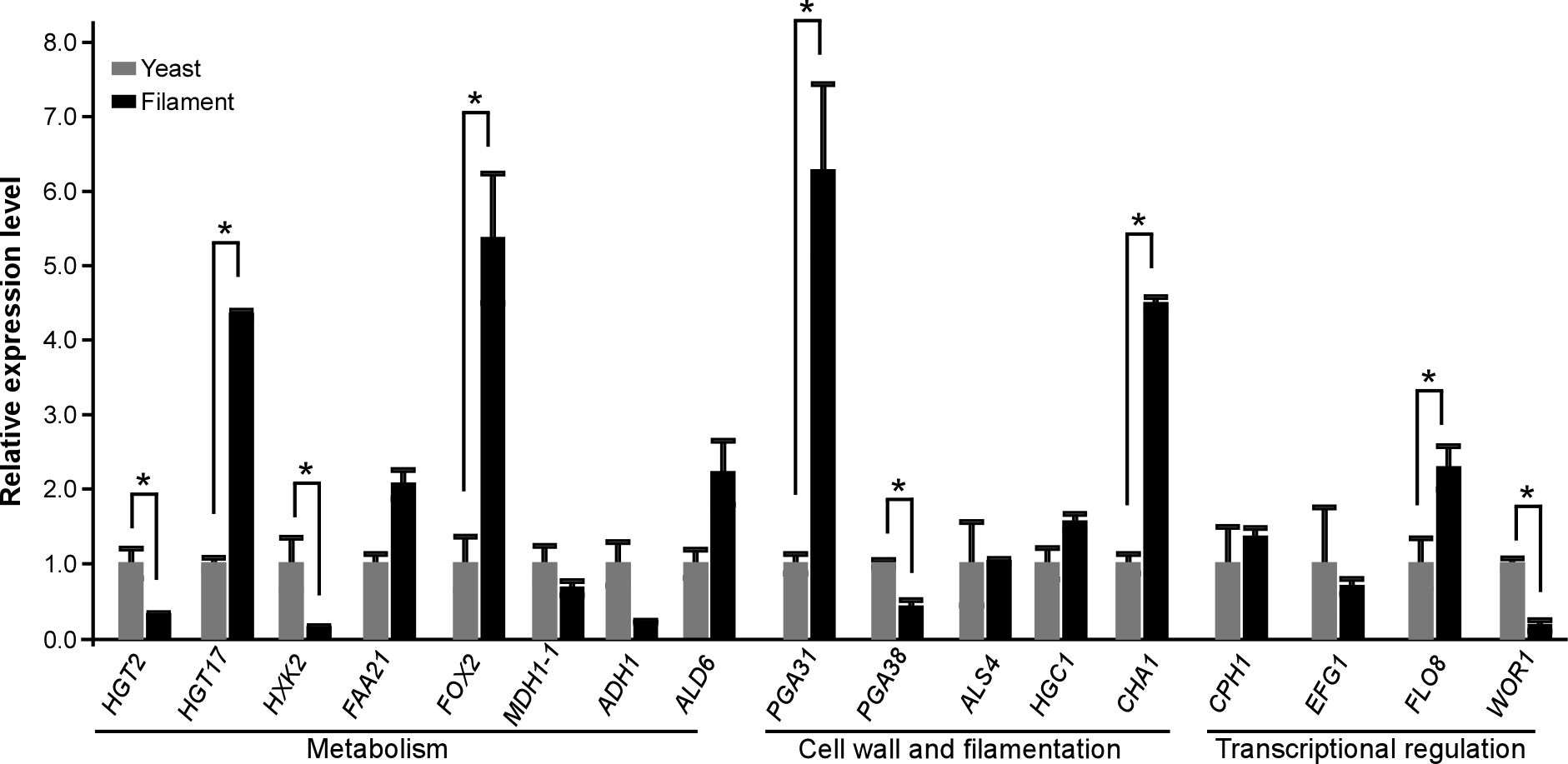

Supplement: Supplementary file 5 — Figure S4 [file 41426_2018_187_MOESM5_ESM.tif]
