# Supplementary material for: Filamentation in Candida auris, an emerging fungal pathogen of humans: passage through the mammalian body induces a heritable phenotypic switch
Source: Emerg Microbes Infect. 2018 Nov 28;7:188. doi: 10.1038/s41426-018-0187-x (PMC6258701; doi:10.1038/s41426-018-0187-x)
Supplement: Supplementary file 6 — Supplementary information [file 41426_2018_187_MOESM6_ESM.docx]

**Supplemental information**

**Figure S1. FM4-64 staining of *C. auris* and *C. albicans* cells.** Typical yeast and filamentous cells of *C. auris* were grown on YPD medium at 25°C for six days. Yeast and filamentous cells of *C. albicans* were grown in liquid YPD and YPD plus 10% serum media, respectively, at 37°C for six hours, and collected for staining assays. White arrows indicate vacuoles. Scale bar represents 10 μm.

**Figure S2. Effects of culture time and temperature on *C. auris* filamentous growth capacity.** Typical yeast (a) or filamentous (b) cells were plated on YPD medium and cultured at 25 or 37°C for three days. Cells continued to be cultured at the initial temperature or switched to the alternative temperature for three additional days of incubation. Representative colonies and cellular images are shown. Percentages of filamentous cells are reported (~1,500 cells were examined for each sample). Scale bar represents 10 μm.

**Figure S3. Morphologies of typical yeast, FC Yeast, and filamentous cells of *C. auris* after 12 to 48 hours of incubation in liquid YPD medium.** 2 x 10^6^ cells/mL of typical yeast, filamentous, and FC yeast cells from YPD medium plates were inoculated into liquid YPD medium and cultured at 37°C or 25°C for 12 to 48 hours. Representative cellular morphologies are shown and percentages of filamentous cells are indicated. Approximately 1,500 cells were examined for the calculation of each percentage. Scale bar represents 10 μm.

**Figure S4. Relative expression levels of selected differentially expressed genes between typical yeast and filamentous cells normalized to *C. auris ACT1*.** Cells of typical yeast or filamentous cells were grown on YPD agar at 37°C for three days and then transferred to 25°C for additional three days. Total RNA was extracted for quantitative RT-PCR assays. The relative expression levels of 17 metabolism-related, cell wall and filamentation-related, and transcriptional regulation-related genes in typical yeast and filamentous cells were examined. The relative expression level of each gene in typical yeast cells was set as “1”. Error bars represent standard errors of two repeats. *, indicates significant difference (p<0.05, two tailed *Student*’s *t*-test).

**Dataset S1. RNA-Seq dataset.** Analysis of differentially expression genes in typical yeast and filamentous cells of *C. auris*.
